# Supplementary material for: The Effects of Competition on Exercise Intensity and the User Experience of Exercise during Virtual Reality Bicycling for Young Adults
Source: Sensors (Basel). 2024 Oct 26;24(21):6873. doi: 10.3390/s24216873 (PMC11548122; doi:10.3390/s24216873)
Supplement: Supplementary file 1 [file sensors-24-06873-s001.zip › Supplemental Figure S1_Intrinsic Motivation Inventory.docx]

**Intrinsic Motivation Inventory (IMI)**

For each of the following statements, please indicate how true it is for you, using the scale:

**I enjoyed doing this activity very much.**

| 1 | 2 | 3 | 4 | 5 | 6 | 7 |
| --- | --- | --- | --- | --- | --- | --- |

Not at all true Somewhat true Very true

**I think I am pretty good at this activity.**

| 1 | 2 | 3 | 4 | 5 | 6 | 7 |
| --- | --- | --- | --- | --- | --- | --- |

Not at all true Somewhat true Very true

**I didn’t try very hard to do well at this activity.**

| 1 | 2 | 3 | 4 | 5 | 6 | 7 |
| --- | --- | --- | --- | --- | --- | --- |

Not at all true Somewhat true Very true

**This activity was fun to do.**

| 1 | 2 | 3 | 4 | 5 | 6 | 7 |
| --- | --- | --- | --- | --- | --- | --- |

Not at all true Somewhat true Very true

**This was an activity that I couldn’t do very well.**

| 1 | 2 | 3 | 4 | 5 | 6 | 7 |
| --- | --- | --- | --- | --- | --- | --- |

Not at all true Somewhat true Very true

**It was important to me to do well at this task.**

| 1 | 2 | 3 | 4 | 5 | 6 | 7 |
| --- | --- | --- | --- | --- | --- | --- |

Not at all true Somewhat true Very true

**I thought this was a boring activity.**

| 1 | 2 | 3 | 4 | 5 | 6 | 7 |
| --- | --- | --- | --- | --- | --- | --- |

Not at all true Somewhat true Very true

**I tried very hard on this activity.**

| 1 | 2 | 3 | 4 | 5 | 6 | 7 |
| --- | --- | --- | --- | --- | --- | --- |

Not at all true Somewhat true Very true

**This activity did not hold my attention at all.**

| 1 | 2 | 3 | 4 | 5 | 6 | 7 |
| --- | --- | --- | --- | --- | --- | --- |

Not at all true Somewhat true Very true

**I put a lot of effort into this.**

| 1 | 2 | 3 | 4 | 5 | 6 | 7 |
| --- | --- | --- | --- | --- | --- | --- |

Not at all true Somewhat true Very true

**I would describe this activity as very interesting.**

| 1 | 2 | 3 | 4 | 5 | 6 | 7 |
| --- | --- | --- | --- | --- | --- | --- |

Not at all true Somewhat true Very true

**I didn’t put much energy into this.**

| 1 | 2 | 3 | 4 | 5 | 6 | 7 |
| --- | --- | --- | --- | --- | --- | --- |

Not at all true Somewhat true Very true

**I thought this activity was quite enjoyable.**

| 1 | 2 | 3 | 4 | 5 | 6 | 7 |
| --- | --- | --- | --- | --- | --- | --- |

Not at all true Somewhat true Very true

**I would be willing to do this again because it has some value to me.**

| 1 | 2 | 3 | 4 | 5 | 6 | 7 |
| --- | --- | --- | --- | --- | --- | --- |

Not at all true Somewhat true Very true

**While I was doing this activity, I was thinking about how much I enjoyed it.**

| 1 | 2 | 3 | 4 | 5 | 6 | 7 |
| --- | --- | --- | --- | --- | --- | --- |

Not at all true Somewhat true Very true

**I believe doing this activity could be beneficial to me.**

| 1 | 2 | 3 | 4 | 5 | 6 | 7 |
| --- | --- | --- | --- | --- | --- | --- |

Not at all true Somewhat true Very true

**Figure S1: Intrinsic Motivation Inventory (IMI).** The IMI consisting of 16 items includes the complete subscales of interest / enjoyment (7 items) and effort / importance (5 items) and partial subscales of value / usefulness (2 items) and competence (2 items).
