# Supplementary material for: The Effects of Competition on Exercise Intensity and the User Experience of Exercise during Virtual Reality Bicycling for Young Adults
Source: Sensors (Basel). 2024 Oct 26;24(21):6873. doi: 10.3390/s24216873 (PMC11548122; doi:10.3390/s24216873)
Supplement: Supplementary file 1 [file sensors-24-06873-s001.zip › Supplemental Table S1.docx]

|  | **Mauchly Test**  (χ^2^) | **Greenhouse-Geisser**  (ε) | **rmANOVA**  (F) |
| --- | --- | --- | --- |
| **Heart Rate (% Max)** | χ^2^ (2) = 15.28  p <0.001 | ε = 0.67 | F(1.33, 30.65) =24.85  p < 0.001, η^2^_partial_ = 0.519 |
| **Raw Cadence (RPM)** | χ^2^ (2) = 20.02  p <0.001 | ε = 0.63 | F(1.26, 30.36) =42.06  p < 0.001, η^2^_partial_ = 0.637 |
| **Normalized Cadence (% faster than baseline)** | χ^2^ (2) = 20.71  p <0.001 | ε = 0.63 | F(1.26, 30.12) =41.57  p < 0.001, η^2^_partial_ = 0.634 |

**Table S1: Omnibus Tests for Exercise Intensity (Aim 1).** Results of the Mauchly test for sphericity, Greenhouse-Geisser corrections, and repeated measured ANOVA are shown for all measures of exercise intensity (Aim 1). Using the Bonferroni-Holm correction to adjust for running 3 repeated measure ANOVAs, there were statistically significant differences for all measures of exercise intensity across the 3 conditions.
