# Supplementary material for: The Effects of Competition on Exercise Intensity and the User Experience of Exercise during Virtual Reality Bicycling for Young Adults
Source: Sensors (Basel). 2024 Oct 26;24(21):6873. doi: 10.3390/s24216873 (PMC11548122; doi:10.3390/s24216873)
Supplement: Supplementary file 1 [file sensors-24-06873-s001.zip › Supplemental Table S2.docx]

|  | **Feedback & Competition Other** | **Feedback &**  **Competition Self** | **Competition Other & Competition Self** |
| --- | --- | --- | --- |
| **Heart Rate (% Max)** | t(24) = -4.74  p < 0.001 | W=284  p < 0.001 | W=118  p > 0.0167 |
| **Raw Cadence (RPM)** | t(24)= -6.04  p < 0.001 | t(24)= -7.53  p < 0.001 | t(24) = -1.92  p >0.0167 |
| **Normalized Cadence (% faster than baseline)** | W=312  p < 0.001 | W=321  p < 0.001 | t(24) = -2.04  p > 0.0167 |

**Table S2:** **Post-Hoc Tests for Exercise Intensity (Aim 1).** Results of the post-hoc tests between conditions for all 3 comparisons are shown for the measures of exercise intensity (Aim 1). T-values are included for comparisons for which the assumption of normality was upheld (paired t-tests) and Wilcoxon signed rank-test scores (W) are shown for comparisons that are not normally distributed.
