# Supplementary material for: The Effects of Competition on Exercise Intensity and the User Experience of Exercise during Virtual Reality Bicycling for Young Adults
Source: Sensors (Basel). 2024 Oct 26;24(21):6873. doi: 10.3390/s24216873 (PMC11548122; doi:10.3390/s24216873)
Supplement: Supplementary file 1 [file sensors-24-06873-s001.zip › Supplemental Table S3.docx]

|  | **Mauchly Test**  (χ^2^) | **Greenhouse-Geisser**  (ε) | **rmANOVA**  (F) |
| --- | --- | --- | --- |
| **Change in RPE**  **(End – Start)** | χ^2^ (2) = 9.33  p =0.009 | ε = 0.74 | F(1.49, 34.18) =31.27  p < 0.001, η^2^_partial_ = 0.576 |
| **Endpoint RPE ( / 20)** | χ^2^ (2) = 16.07  p < 0.001 | ε = 0.67 | F(1.33, 31.9) = 34.03  p < 0.001, η^2^_partial_ = 0.586 |
| **IMI Effort ( / 7)** | χ^2^ (2) = 19.88  p <0.001 | ε = 0.63 | F(1.27, 30.41) =14.33  p < 0.001, η^2^_partial_ = 0.374 |
|  | | | |
| **IMI Enjoyment ( / 7)** | χ^2^ (2) = 2.38  p >0.05 | NA | F(2, 48) = 3.83  p = 0.029, η^2^_partial_ = 0.138 |
| **IMI Total ( / 7)** | χ^2^ (2) = 11.97  p = 0.003 | ε = 0.71 | F(1.42, 34.15) = 10.48  p < 0.001, η^2^_partial_ = 0.304 |

**Table S3: Omnibus Tests for User Experience of Exercise (Aim 2).** Results of the Mauchly test for sphericity, Greenhouse-Geisser corrections, and repeated measured ANOVA are shown for all measures of the user experience of exercise (Aim 2). Using the Bonferroni-Holm correction to adjust for running 5 repeated measure ANOVAs, there were statistically significant differences for all measures of the user experience of exercise across the 3 conditions. Of note, alpha = 0.05 for IMI enjoyment.
