# Supplementary material for: The Effects of Competition on Exercise Intensity and the User Experience of Exercise during Virtual Reality Bicycling for Young Adults
Source: Sensors (Basel). 2024 Oct 26;24(21):6873. doi: 10.3390/s24216873 (PMC11548122; doi:10.3390/s24216873)
Supplement: Supplementary file 1 [file sensors-24-06873-s001.zip › Supplemental Table S4.docx]

|  | **Feedback & Competition Other** | **Feedback &**  **Competition Self** | **Competition Other & Competition Self** |
| --- | --- | --- | --- |
| **Change in RPE**  **(End – Start)** | t(23)= -5.15  p < 0.001 | t(23) = -6.79  p < 0.001 | t(24) = -2.16  p > 0.0167 |
| **Endpoint RPE ( / 20)** | W= 286.0  p < 0.001 | W = 291.0  p < 0.001 | W= 62.0  p > 0.0167 |
| **IMI Effort ( / 7)** | W=258.5  p = 0.002 | W=243  p = 0.001 | W=39.5  p >0.0167 |
|  | | | |
| **IMI Enjoyment ( / 7)** | t(24) = -1.60  p > 0.0167 | W = 32.5  p = 0.007 | W = 81.5  p >0.0167 |
| **IMI Total ( / 7)** | t(24) = -3.05  p = 0.006 | t(24) = -3.73  p = 0.001 | t(24) = -1.44  p > 0.0167 |

**Table S4:** **Post-Hoc Tests for User Experience of Exercise (Aim 2).** Results of the post-hoc tests between conditions for all 3 comparisons are shown for the measures of user experience of exercise (Aim 2). T-values are included for comparisons for which the assumption of normality was upheld (paired t-tests) and Wilcoxon signed rank-test scores (W) are shown for comparisons that are not normally distributed.
