# Supplementary material for: The Effects of Competition on Exercise Intensity and the User Experience of Exercise during Virtual Reality Bicycling for Young Adults
Source: Sensors (Basel). 2024 Oct 26;24(21):6873. doi: 10.3390/s24216873 (PMC11548122; doi:10.3390/s24216873)
Supplement: Supplementary file 1 [file sensors-24-06873-s001.zip › Supplemental Table S5.docx]

|  | **Mauchly Test**  (χ^2^) | **Greenhouse-Geisser**  (ε) | **rmANOVA**  (F) |
| --- | --- | --- | --- |
| **Task Focus (%)** | χ^2^ (2) = 9.92  p = 0.007 | ε = 0.73 | F(1.45, 31.97) =3.32  p = 0.063, η^2^_partial_ = 0.131 |
| **Roadside Gazes (%)** | χ^2^ (2) = 23.54  p < 0.001 | ε = 0.60 | F(1.20, 26.29) = 4.32, p = 0.041, η^2^_partial_ = 0.164 |
| **Rightward Gazes (%)** | χ^2^ (2) = 28.35  p <0.001 | ε = 0.57 | F(1.15, 25.28) =3.69  p = 0.061, η^2^_partial_ = 0.144 |
| **Water Gazes (%)** | (χ^2^ (2) = 16.24, p <0.001 | ε = 0.65 | F(1.30, 28.60) = 5.86  p = 0.016, η^2^_partial_ = 0.210 |

**Table S5: Omnibus Tests for Measures of Visual Attention (Aim 3).** Results of the Mauchly test for sphericity, Greenhouse-Geisser corrections, and repeated measured ANOVA are shown for all measures of visual attention (Aim 3). Using the Bonferroni-Holm correction to adjust for running 4 repeated measure ANOVAs, there were no statistically significant differences in any of the measures of visual attention across the 3 conditions. For water gazes, alpha = 0.0125. For roadside gazes, alpha = 0.025. For rightward gazes, alpha = 0.0375. For task focus, alpha = 0.05.
