# Supplementary material for: The Effects of Competition on Exercise Intensity and the User Experience of Exercise during Virtual Reality Bicycling for Young Adults
Source: Sensors (Basel). 2024 Oct 26;24(21):6873. doi: 10.3390/s24216873 (PMC11548122; doi:10.3390/s24216873)
Supplement: Supplementary file 1 [file sensors-24-06873-s001.zip › Supplemental Table S6.docx]

|  | **Feedback & Competition Other** | **Feedback &**  **Competition Self** | **Competition Other & Competition Self** |
| --- | --- | --- | --- |
| **Task Focus (%)** | NA | NA | NA |
| **Roadside Gazes (%)** | W = 71.0  p = 0.042 | W = 31.0  p = 0.002 | W = 166.0  p >0.0167 |
| **Rightward Gazes (%)** | NA | NA | NA |
| **Water Gazes (%)** | W = 36.0  p = 0.031 | W = 27.0  p = 0.011 | W = 73.0  p > 0.0167 |

**Table S6:** **Post-Hoc Tests for Measures of Visual Attention (Aim 3).** Results of the post-hoc tests between conditions for all 3 comparisons are shown for the measures of visual attention (Aim 3). T-values are included for comparisons for which the assumption of normality was upheld (paired t-tests) and Wilcoxon signed rank-test scores (W) are shown for comparisons that are not normally distributed.
